# Baicalein decreases uric acid and prevents hyperuricemic nephropathy in mice

**DOI:** 10.18632/oncotarget.16928

**Published:** 2017-04-07

**Authors:** Xiaolu Meng, Zhuo Mao, Xin Li, Dandan Zhong, Min Li, Yingli Jia, Jing Wei, Baoxue Yang, Hong Zhou

**Affiliations:** ^1^ Department of Pharmacology, School of Basic Medical Sciences, Peking University, Beijing, 100191, P.R. China; ^2^ Tianjin Key Laboratory for Modern Drug Delivery and High-Efficiency, School of Pharmaceutical Science and Technology, Tianjin University, Tianjin, 300072, P.R. China; ^3^ Drug Clinical Trial Institution, The Affiliated Hospital of Qingdao University, Qingdao, 266003, P.R. China

**Keywords:** baicalein, hyperuricemia, uric acid, nephropathy, xanthine oxidoreductase

## Abstract

Baicalein, a natural flavonoid, is structurally advantageous for binding to xanthine oxidoreductase. In our study, molecular docking analysis and Surface Plasmon Resonance revealed a direct interaction between baicalein and xanthine oxidoreductase. Moreover, 50 mg/kg/d baicalein treatment significantly suppressed the viability of xanthine oxidoreductase in hyperuricemia mouse model. The data showed that baicalein remarkably prevented renal dysfunction, ameliorated kidney fibrosis, alleviated epithelial-mesenchymal transition and oxidative stress in hyperuricemia mice. Thus, we concluded that baicalein executed a kidney-protection action in hyperuricemia and therefore may be used as a therapeutic alternative for hyperuricemic nephropathy.

## INTRODUCTION

Hyperuricemia is an independent risk factor for kidney diseases, metabolic syndrome, diabetes, hypertension and cardiovascular diseases [1–4]. Moreover, high-uric acid (UA) induces cognitive dysfunction through hippocampal inflammation in rodents and humans [5]. Also, hyperuricemia increases the 1-year mortality of ST-segment elevation myocardial infarction (STEMI) patients in Killip class I [6]. Besides, the control of serum UA can prevent tumor lysis syndrome, which is a life-threatening oncologic emergency[7].

Because approximately 70% of UA is excreted from the kidney, hyperuricemia occurs when kidney function declines [8]. However, the role of elevated UA on chronic kidney diseases (CKD) remains controversial. Notably, elevated UA level induces renal dysfunction in a great deal of animal studies, but for humans, the relationship between UA and kidney disease is not that simple [9]. UA levels can be changed by other risk factors, including hypertension, metabolic syndrome and microalbuminuria, but it is not clear whether these are mediators or confounders. Most epidemiological evidence suggests a direct link between UA and CKD, but which comes first cannot be determined [10]. Therefore, there is no evidence to definitely demonstrate whether UA is causal, compensatory, coincidental or it is only an epiphenomenon in these patients [11]. At present, urate-lowering therapy in asymptomatic hyperuricemia is also conservative [12–14]. Thus, further experimental and clinical studies are still needed to identify the association between hyperuricemia and CKD.

Hyperuricemia is result from overproduction from hepatic metabolism and cell turnover or renal underexcretion. Urate is formed from dietary purines and endogenously synthesized purines. Urate is formed in the liver, where xanthine oxidoreductase (XOR) at play. XOR is a prototypical molybdenum hydroxylase, catalyzing hydroxylation of hypoxanthine to xanthine as well as xanthine to uric acid [15]. This reaction occurs at a molybdenum-pterin center. From there, the electrons are transferred via two Fe_2_S_2_ clusters to the isoalloxazine ring of flavin. Then flavin passes them on to the second substrate NAD^+^ [16]. Thus, inhibiting XOR helps downregulate UA production. As the prevalence of hyperuricemia is increasing globally, new treatment options to manage hyperuricemia is demanded. Currently, urate-lowering drugs contain three main classes: XOR inhibitors, uricosurics, URAT1 inhibitor, and recombinant uricases [17]. Among them, allopurinol, a XOR inhibitor, is first-line therapy [18]. However, it presents unfavorable side effects clinically, so does other medicines. So non-purine XOR inhibitors with less toxicity grasp stronger interest. Moreover, many Chinese medical herbs are significantly superior to Western medicine in overall efficacy with fewer adverse drug reactions [19].

As a natural flavonoid extracted from the Chinese herb *Scutellariae radix*, baicalein has many biochemical and pharmacological benefits, including antioxidant [20], anti-inflammation [21, 22], anti-tumor [23], anti-fibrosis and cardiovascular protective effects [24, 25]. What's more, baicalein can improve cardiac function [26], attenuate neurological deficits [27], treat gastric ulcers [28] *etc*. Given these data, we hypothesize that baicalein can prevent hyperuricemia and alleviate kidney damage. As baicalein exhibits strong first-pass metabolism in small intestine, we administrated baicalein 6,7-biacetate instead in our mouse model. It partly prevents glucuronidation and degrades into baicalein in intestinal track after oral administration. In our study, mouse hyperuricemia model and molecular pharmacology methods were exerted to investigate UA-lowering and renal protective effects of baicalein.

## RESULTS

### Identification of the interaction between baicalein and XOR

**Figure 1:**
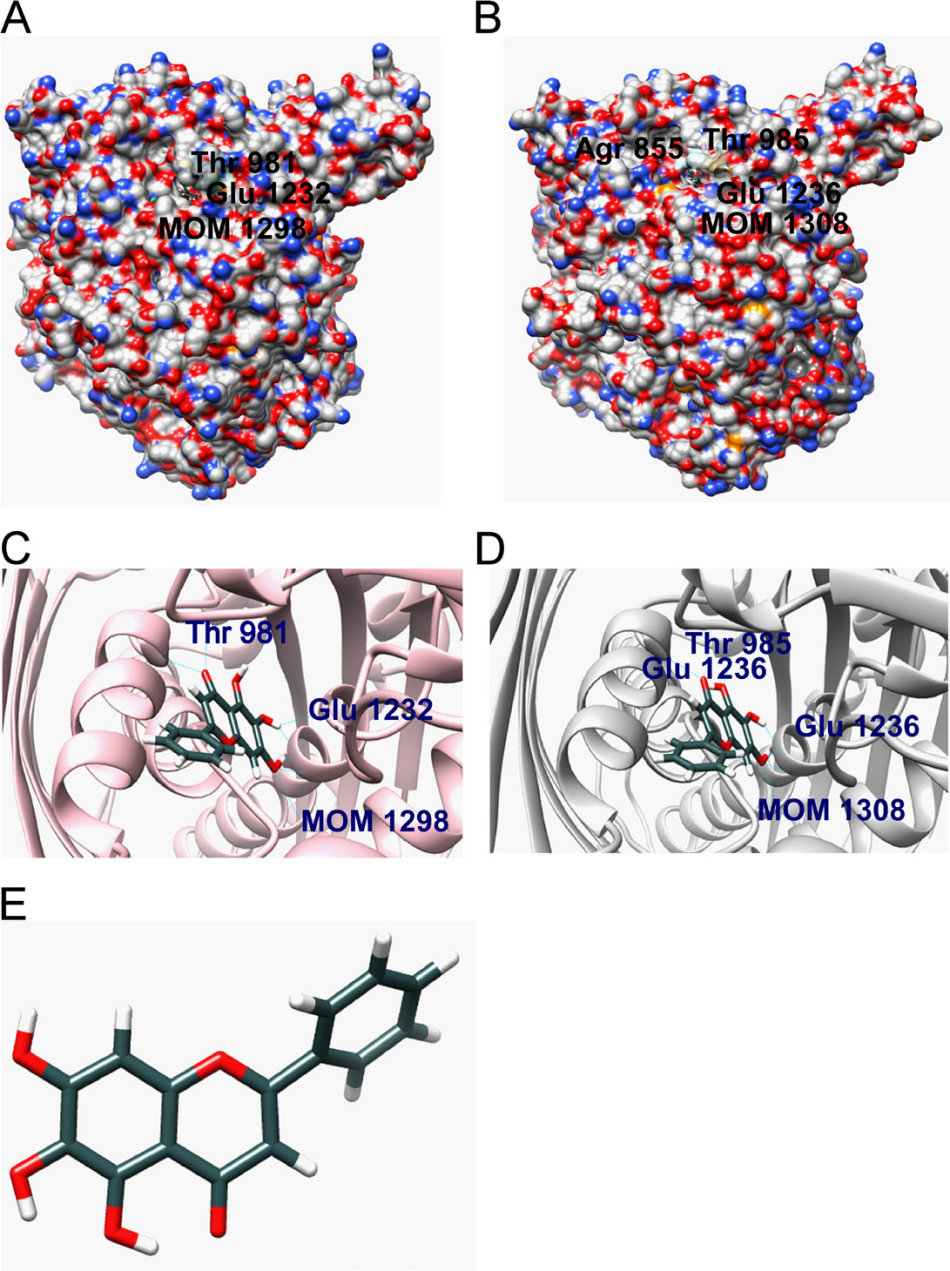
Molecular docking (**A**) The binding model of rat source XOR-baicalein complex (XOR is shown using a surface model and baicalein is shown using a ball and stick model). (**B**) The binding model of human source XOR-baicalein complex (XOR is shown using a surface model and baicalein is shown using a ball and stick model). (**C**) The insight model of rat source XOR-baicalein complex. (**D**) The insight model of human source XOR-baicalein complex. (**E**) Molecular structure of baicalein.

Molecular docking method is used for constructing receptor-ligand complex and binding mechanism analysis. The binding modes of baicalein with rat source and human source XOR were performed and they both presented similar conformation in enzyme binding pocket (Figure 1A–D). For rat source XOR-baicalein system, Glu1261, Thr1010 of XOR binding pocket provided hydrogen bonds interaction with baicalein; Leu1011, Phe1009, Phe914, Ala1079, and Ala1078 made hydrophobic interactions with baicalein; Phe914 and Phe1009 were involved in π-cation interaction with aromatic ring of baicalein. Meanwhile, similar contributions also existed in human source XOR-baicalein system. Amino acid residues of XOR binding pocket providing hydrogen bonds included Arg881, Thr1010, and Glu1262; hydrophobic interactions were made via Phe1010, Phe915, Ala1080, Ala1079, Val808, and Val1012; Phe915 and Phe1010 participated in π-cation interaction. The details of hydrogen bonds were shown in Tables 1 and 2.

**Table 1:** Distances (Å) between hydrogen bond donor and acceptor in rat source XOR-baicalein complex

H-bond donor	H-bond acceptor	Distances (Å)
Baicalein O4	Glu1232 OE1	3.078
Baicalein O4	Glu1232 OE2	3.019
Baicalein O5	Glu1232 OE2	2.805
Thr981 N	Baicalein O2	2.520
Thr981 OG1	Baicalein O2	2.647
MOM OM1	Baicalein O5	1.963
MOM OM3	Baicalein O5	3.262

**Table 2:** Distances (Å) between hydrogen bond donor and acceptor in human source XOR-baicalein complex

H-bond donor	H-bond acceptor	Distances (Å)
Baicalein O4	Glu1236 OE1	3.298
Baicalein O5	Glu1236 OE2	2.842
Thr985 N	Baicalein O2	2.654
Thr985 OG1	Baicalein O2	2.703
Arg855 NE	Baicalein O3	2.766
Arg855 NH2	Baicalein O3	2.809
MOM OM1	Baicalein O5	1.765
MOM OM3	Baicalein O5	3.435

### The binding affinity of baicalein to XOR based on Surface Plasmon Resonance (SPR) biosensor analysis

**Figure 2:**
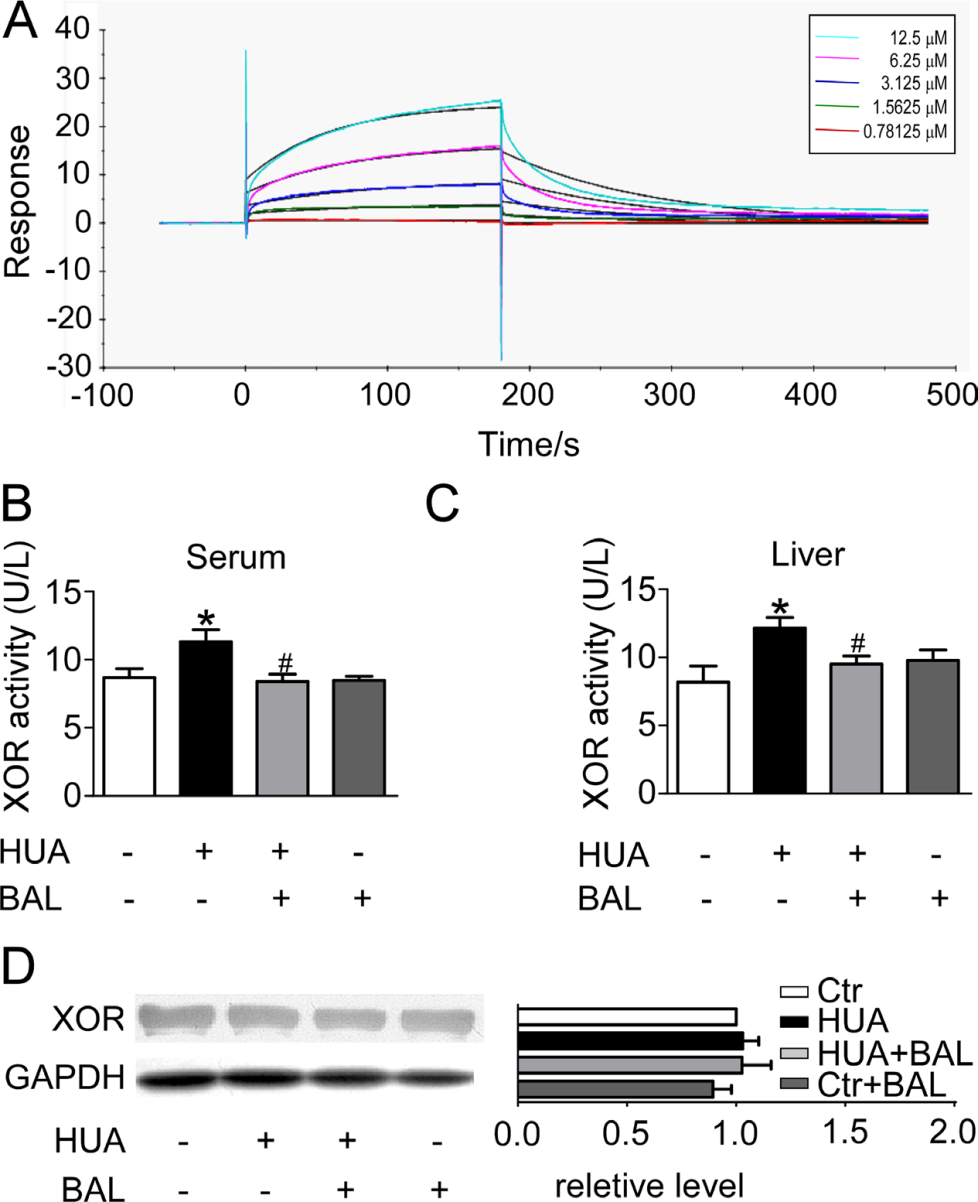
Binding affinity of baicalein to XOR as determined by SPR, and the inhibitory effect of baicalein to XOR in hyperuricemia mice *in vivo* (**A**) Real-time measurements of the binding affinity of baicalein to XOR were performed using a Biacore 3000 instrument. Baicalein at a concentration of 0.78125, 1.5625, 3.125, 6.25 or 12.5 μM (curves from bottom to top) was injected. (**B**) ICR male mice were intraperitoneally administered with hypoxanthine and potassium oxonate daily to establish hyperuricemia model. Baicalein was orally given to some mice. Blood samples were collected for evaluating enzymatic activity of XOR. (**C**) Liver tissues were homogenized for evaluating enzymatic activity of XOR. Values were means ± SEM (*n* = 5–6). *P < 0.05 vs. control group. ^#^*P* < 0.05 vs. hyperuricemia group. (**D**) Expression levels of XOR in liver were determined by western blots (left) and quantifications (right), which were normalized with GAPDH. Means ± SEM (*n* = 3).

To verify the prediction from computational docking analysis, the binding affinity of baicalein to XOR was determined by SPR biosensor technology. Biacore 3000 instrument was utilized to record the ability of baicalein binding to XOR, which was reflected by the RU values. The RU increased as baicalein concentration was elevated, indicating that baicalein bound to XOR in a concentration-dependent manner (Figure 2A). The association (K_on_), dissociation (K_off_), and equilibrium dissociation constants (K_D_) of baicalein binding to XOR were 607.7 M^-1^S^-1^, 9.467 × 10^–3^ S^-1^, and 6.749 × 10^–5^ M, respectively. The results showed that baicalein bound exactly to XOR.

### Baicalein inhibited the enzymatic activity while had no influence on the expression of XOR

In the established hyperuricimic mouse model, the activity of XOR in liver and in serum were both elevated. Treatment with 50 mg/kg baicalein 6,7-biacetate per day effectively downregulated XOR activity both in liver and in serum (Figure 2B, 2C). However, baicalein has no significant influence on XOR expression in liver (Figure 2D).

### Baicalein lowered UA and protected kidney against hyperuricemia

**Figure 3:**
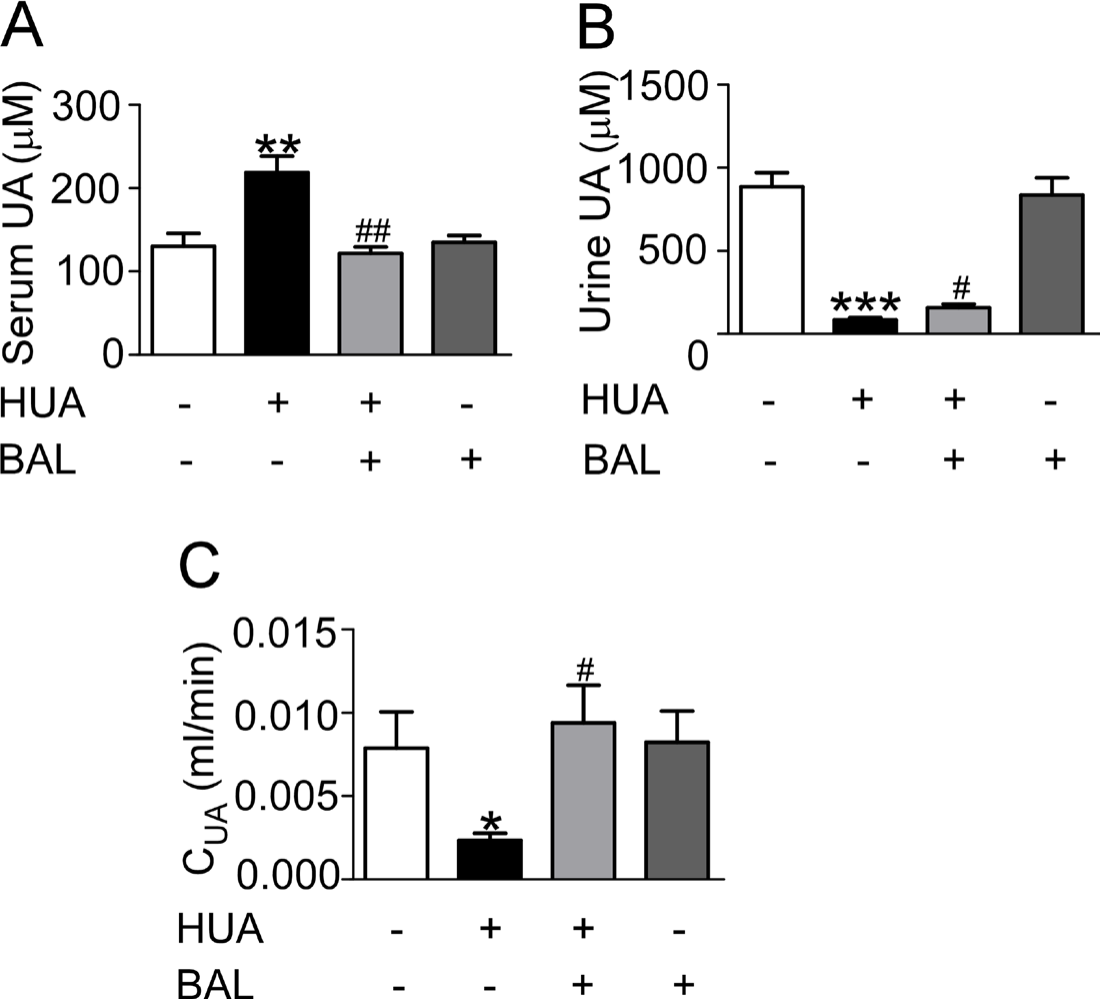
Baicalein reduced UA level of hyperuricemia mice *in vivo* Blood and urine were collected and analyzed after hypoxanthine and potassium oxonate treatment for 2 weeks. (**A**) Serum UA. (**B**) Urine UA. (**C**) UA clearance. C_UA_ (ml/min) = urine UA (μM) × urine output (ml) / t (min)/ serum UA (μM). Values were means ± SEM (*n* = 5–6). *P < 0.05, **P < 0.01 and ***P < 0.001 vs. control group. ^#^*P* < 0.05, ^##^*P* < 0.01 vs. hyperuricemia group.

**Figure 4:**
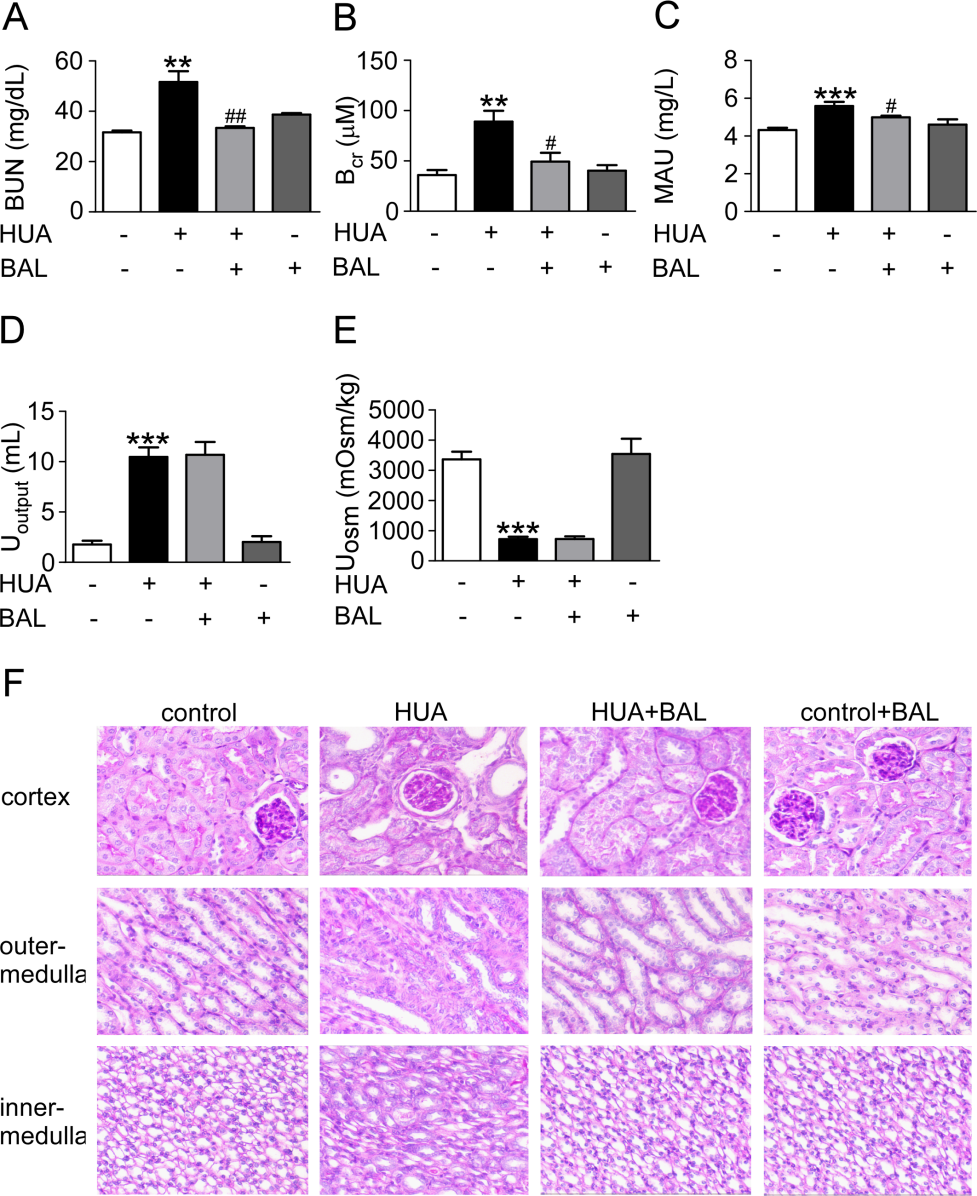
Baicalein improved renal function and kidney pathology in hyperuricemia mice Blood, urine and kidney samples were collected for renal function tests and histological examination. (**A**) BUN. (**B**) Blood creatinine. (**C**) Urine microalbumin. (**D**) Urine output. (**E**) Urine osmolality. (**F**) Photomicrographs illustrated periodic acid–Schiff staining of the kidney tissues in control or hyperuricemia mice with or without baicalein treatment (magnification 200×). Values were means ± SEM (*n* = 5–6). *P < 0.05, **P < 0.01 and ***P < 0.001 vs. control group. ^#^*P* < 0.05, ^##^*P* < 0.01 vs. hyperuricemia group.

Renal function was assessed by serum UA (Figure 3A), urine UA (Figure 3B), UA clearance (Figure 3C), BUN (Figure 4A), serum creatinine (Figure 4B), proteinuria (Figure 4C), urine output (Figure 4D) and osmalility (Figure 4E). As shown in Figures 3 and 4, serum UA, creatinine, BUN and urinary proteinuria were all elevated in hyperuricemia mice compared with control mice, suggesting that mice develop hyperuricemic nephropathy in this model. Besides, UA clearance was significantly decreased in hyperuricemia mice (Figure 3C). Treatment with baicalein 50 mg/kg/day for 21 days improved renal function notably. As the trend of urinary osmolality was contrary to that of urine output in each group, urine concentrating ability was normal in hyperuricemia mice (Figure 4D, 4E). Furthermore, Periodic acid–Schiff staining showed that kidneys of hyperuricemia mice developed severe tubulointerstitial damage with tubular dilatation and interstitial fibrosis. In outer-medulla, we saw protein casts in dilated tubules and slight interstitial collagen accumulation. Moreover, the inner-medulla presented tubular dilatation also and the epithelial cells were disarranged. Baicalein administration preserved kidney architecture and moderated the tubulointerstitial damage (Figure 4F). Thus, baicalein can improve renal function and alleviate kidney injury in hyperuricemia.

### Baicalein modified XOR-dependent and NADPH oxidase-dependent renal oxidative stress in hyperuricemia mice

**Figure 5:**
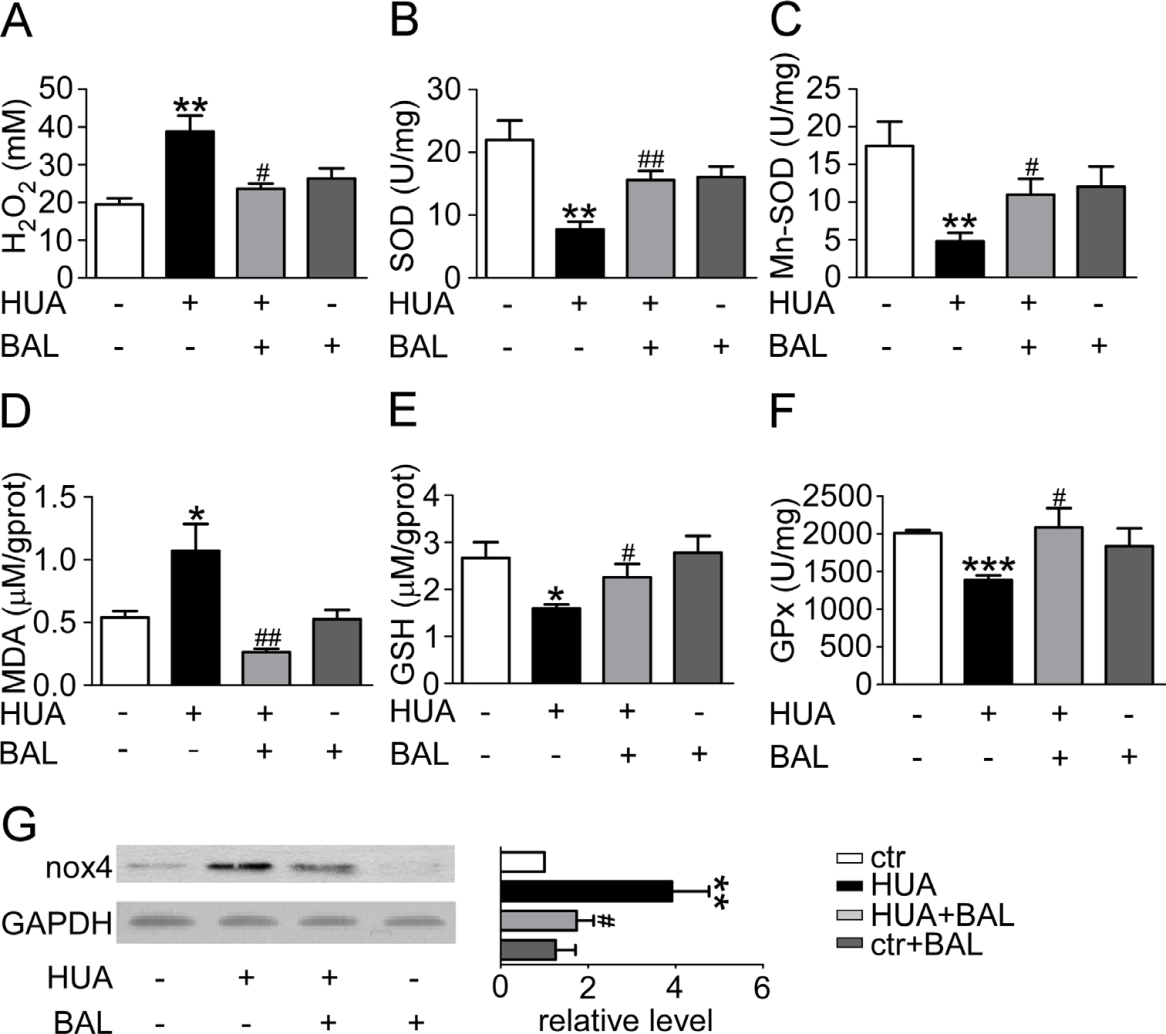
Baicalein prevented renal oxidative stress in hyperuricemia mice Blood was collected to determine ROS level and kidney tissues were homogenized for evaluating the levels of different enzymes. (**A**) H_2_O_2_ level of mouse blood. (**B**) SOD activity in renal tissue. (**C**) Mn-SOD activity in renal tissue. (**D**) Malondialdehyde activity in renal tissue. (**E**) GSH level in renal tissue. (**F**) GPx activity in renal tissue. Means ± SEM (*n* = 5–6). *P < 0.05, **P < 0.01 and ***P < 0.001 vs. control group. ^#^*P* < 0.05, ^##^*P* < 0.01 and ^###^*P* < 0.001 vs. hyperuricemia group. (**G**) Expression levels of Nox4 in kidney were determined by western blots (left) and quantifications (right), which were normalized with GAPDH. Means ± SEM (*n* = 4).

XOR generates oxidative stress while the oxidative hydroxylation of xanthine to uric acid takes place. Serum H_2_O_2_ level was elevated in hyperuricemia mice and baicalein downregulated its concentration (Figure 5A). Malondialdehyde, superoxide dismutase (SOD), reduced glutathione (GSH) and glutathione peroxidase (GPx) were detected to evaluate the effect of baicalein on XOR-mediated oxidative stress in kidneys. Compared with the sham group, SOD (Figure 5B) and Mn-SOD (Figure 5C) level were suppressed while Malondialdehyde (Figure 5D) level was greatly elevated in hyperuricemia mice. GSH (Figure 5E) and GPx (Figure 5F) were also downregulated in hyperuricemia mouse. We found that 21 days of baicalein treatment at 50 mg/kg/day reversed the situation effectively. Meanwhile, we evaluate the change of NADPH oxidase 4 (Nox4), which expressed abundantly in renal proximal tubule, and fount it upregulated in hyperuricemia mice while downregulated by baicalein (Figure 5G). These results indicated that baicalein modified XOR-dependent and NADPH oxidase-dependent renal oxidative stress in hyperuricemia mice.

### Baicalein suppresses hyperuricemia-induced renal fibrosis through matrix metalloproteinases (MMPs)

**Figure 6:**
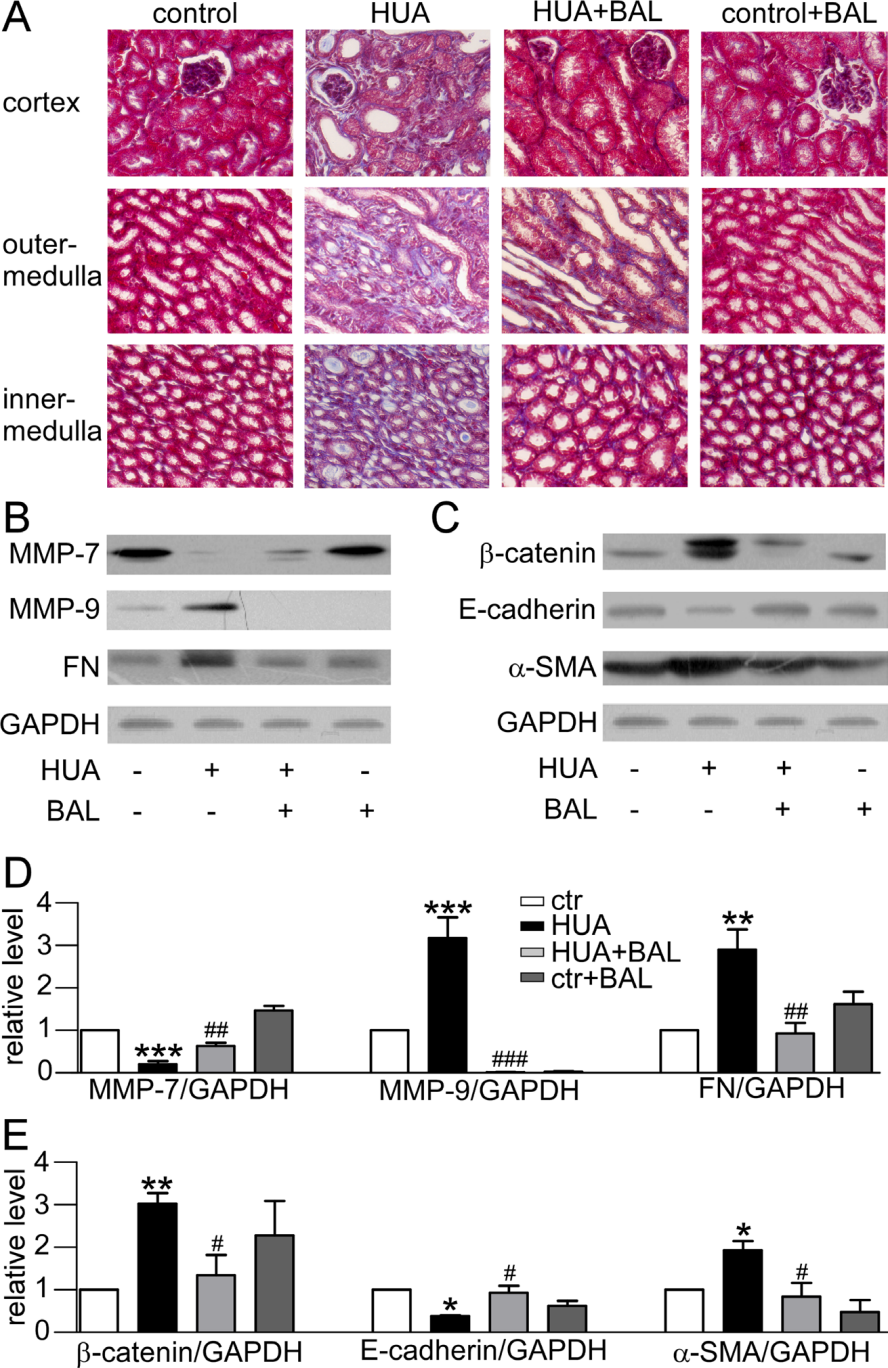
Effect of baicalein on fibrosis and EMT related proteins in mice (**A**) Photomicrographs illustrated Masson trichrome staining of the kidney tissues in control or hyperuricemia mice with or without baicalein treatment (magnification 200×). (**B**) Western blots of fibrosis-related protein. (**C**) Western blots of EMT-related protein. (**D**) Quantification of fibrosis-related protein. (**E**) Quantification of EMT-related protein. The data were normalized by the intensity of GAPDH. Means ± SEM (*n* = 4). *P < 0.05, **P < 0.01 and ***P < 0.001 vs. control group. ^#^*P* < 0.05, ^##^*P* < 0.01 and ^###^*P* < 0.001 vs. hyperuricemia group.

Masson trichrome stain demonstrated that baicalein could moderate hyperuricemia induced renal fibrosis. Hyperuricemic mouse kidneys displayed severe morphologic lesions. It is characterized by tubular dilation with epithelial atrophy and interstitial expansion with collagen accumulation. Treatment with baicalein showed a remarkable improvement of the morphologic lesions with less fibrosis in interstitium (Figure 6A). At the same time, the expression of fibronectin, a key component of the interstitial matrix, was upregulated in hyperuricemia mice and downregulated by baicalein (Figure 6B). As MMPs were closely linked to fibrosis and changed a lot in our RNA-seq data (data not shown), we detected MMP-7 and MMP-9 level in mouse kidneys. They were downregulated and upregulated in hyperuricemia model respectively. Baicalein significantly reversed their expression (Figure 6B). Meanwhile, β-catenin level was elevated in hyperuricemia mice (Figure 6C). Therefore, baicalein may suppress hyperuricemia-induced renal fibrosis involving MMP-7 and MMP-9 signals.

### Baicalein inhibits hyperuricemia-induced epithelial-mesenchymal transition (EMT) process

EMT markers were detected in our study. In hyperuricemia mouse model, the expression of E-cadherin was suppressed while α-smooth muscle actin (α-SMA) was elevated remarkably, indicating EMT process. Treatment with baicalein 50 mg/kg/day blocked this pathway (Figure 6C).

## DISCUSSION

In this paper, we firstly identified baicalein as an effective XOR inhibitor *in vitro* and *in vivo*. XOR is a highly versatile molybdoflavin enzyme, ubiquitous among species (from bacteria to human) and within the various tissues of mammals [15]. It catalyzes the production of UA, and is also a significant source of reactive species [29]. Thus, human XOR is a drug target for hyperuricemia treatment, and potentially for relevant diseases as well [30].

The active cavity of XOR is a long, narrow channel leading to the active site [31]. If the cavity is bound by an inhibitor, the channel and surrounding space are mostly plugged. It will hold up the landing of xanthine (substrate) and ultimately preventing its oxidation [31]. Natural products including flavonoids exhibit diversified bioactivities and lower toxicity compared with classical XOR inhibitors [32]. Flavonoids have a potent inhibitive effect towards XOR both *in vitro* and *in vivo* [33], such as apigenin, luteolin, hyperin *etc* [34]. C2 = C3 double bonds of flavonoids contributed to their planar structure and the hydroxyl groups on C-5 and C-7 were advantageous for binding to XOR [34]. Baicalein is a natural flavonoid with all these structure superiorities above. Thus, the possibility that baicalein can inhibit XOR *in vivo* and fight against hyperuricemia cannot be excluded. Computational docking analysis indicated that baicalein was located in the binding pocket and interacted with several amino acid residues so as to plug the enzyme cavity. To verify the interaction between baicalein and XOR, we further evaluated their binding affinity using SPR biosensor analysis. The results confirmed the computer modeling prediction. In terms of mice and human, XOR level was highest in liver and intestine. Besides, a wide range of activity levels were in biological fluids, such as blood [35]. So we tested XOR level of liver and serum in hyperuricemia mouse model *in vivo*. XOR viability in liver and serum both elevated, but baicalein significantly suppressed the viability of XOR. Interestingly, the expression of XOR in liver was not significantly changed after treatment with baicalein. We tested XOR expression in kidney also, but it was too low to be detected (data not shown). Thus, we concluded that baicalein conducted a uric acid-lowering effect through inhibiting XOR viability while not affecting its expression.

XOR acts as not only a producer of UA, but a major producer of reactive oxygen species (ROS) [32]. XOR releases O_2_^–^ and H_2_O_2_, which could be converted to more toxic ROS, peroxynitrate (ONOO^–^), hydroxyl anion (OH^–^), and hypochorous acid (HOCl). They all do harm to proteins, lipids, carbohydrates, DNA, RNA, subcellular organelles and cell systems [36]. XOR generates ~25% O_2_^–^ and ~75% H_2_O_2_ while the oxidative hydroxylation of xanthine to UA takes place [37]. So we detected H_2_O_2_ level and saw an increase in hyperuricemia mice and baicalein renormalized H_2_O_2_ concentration.

Oxidative stress is a disease mechanism common to a variety of disorders harming human health [39]. It can result in DNA damage, lipid peroxidation, protein modification, and other pathological effects in various chronic disorders, including neurodegenerative, cardiovascular and renal diseases, and cancer [40–43]. In organism, ROS levels are kept within a narrow range by enzymatic antioxidants, such as SOD, GPx, or nonenzymatic antioxidants, such as ascorbic acid, GSH [44]. Baicalein scavenged hydroxyl free radicals, O_2_^–^, diphenylpicrylhydrazyl free radicals, and lipid peroxyl radicals [45]. Also, baicalein could inhibit H_2_O_2_ inducing RAW264.7 apoptosis, hypodiploid generation, DNA breakage, caspase-3 activation and cytochrome C release [46]. In our study, an increase of H_2_O_2_ level and a decrease of SOD, GPx and GSH activity were observed. Pretreatment of baicalein significantly reversed the situation. At the same time, hyperuricemia induced activation of NADPH oxidase and it was inhibited by baicalein. These results indicate that baicalein may block both of the XOR-dependent and NADPH oxidase-dependent production of ROS and enhance ROS elimination. This normalizes the imbalance between the oxidative and anti-oxidative status after hyperuricemia.

In addition to oxidative stress XOR brought about, we cannot ignore the damage caused by its final production—UA. Because of excessive UA production and decreased UA clearance, hyperuricemia is a common finding in CKD [8]. As baicalein inhibited XOR effectively (Figures 1, 2) while had no influence on renal transporters (data not shown), baicalein possibly decreased UA level mainly through suppression of UA production. Although UA is a major antioxidant in human blood that may protect against aging and oxidative stress, it also causes or exacerbates inflammation, endothelial dysfunction, mesangial and fibroblast activation, and finally kidney fibrosis and progressive CKD [47–49]. Furthermore, UA may cause hypertension and metabolic syndrome, which are thought to be initiated by oxidative stress [50, 51]. In line with these studies, we found micro-albuminuria in hyperuricemia mice, which is a well-known early marker of CKD. And kidneys of hyperuricemia mice displayed severe morphologic lesions characterized by tubular dilation and interstitial collagen accumulation. So we focused on renal fibrosis and relevant pathways.

As MMPs greatly changed in our RNA-seq results (data not shown), we investigated their alterations in hyperuricemia. MMPs is a large family of zinc-dependent endopeptidases. They are collectively capable of proteolyzing all components of the extracellular matrix (ECM). MMPs in the pathogenesis of nephropathy is controversial and inconsistent [52]. They regulate fibrotic process either positively or negatively via targeting different sets of substrate proteins for degradation [53]. Interestingly, our data showed an elevated-MMP-9 level and a decreased-MMP-7 level in hyperuricemia mouse. This result is according with a wide variety studies recognizing MMP-9 as a biomarker of renal injury [52, 54]. In terms of MMP-7, it may be more complicated. This trend is in line with elevated expression of fibronectin, which presents as the cleavage substrate of MMP-7. Apart from that, we blamed it to the overcapacity of ROS. The MMPs are synthesized as inactive zymogens maintained by a conserved cysteine residue. It forms Cys-Zn^2+^ coordination and prevents water molecules from catalysis [55]. However, the cysteine switch compasses a thiol residue. It can be altered by ROS and results in dissociation from the catalytic site and enzyme activation. So MMP-7 can be activated at lower concentrations of ROS. However, at a higher concentration of ROS, MMP-7 is inactivated by oxidizing specific amino acid residues. It affects the conformine of active site, possibly preventing overactivity [52, 56].

**Figure 7:**
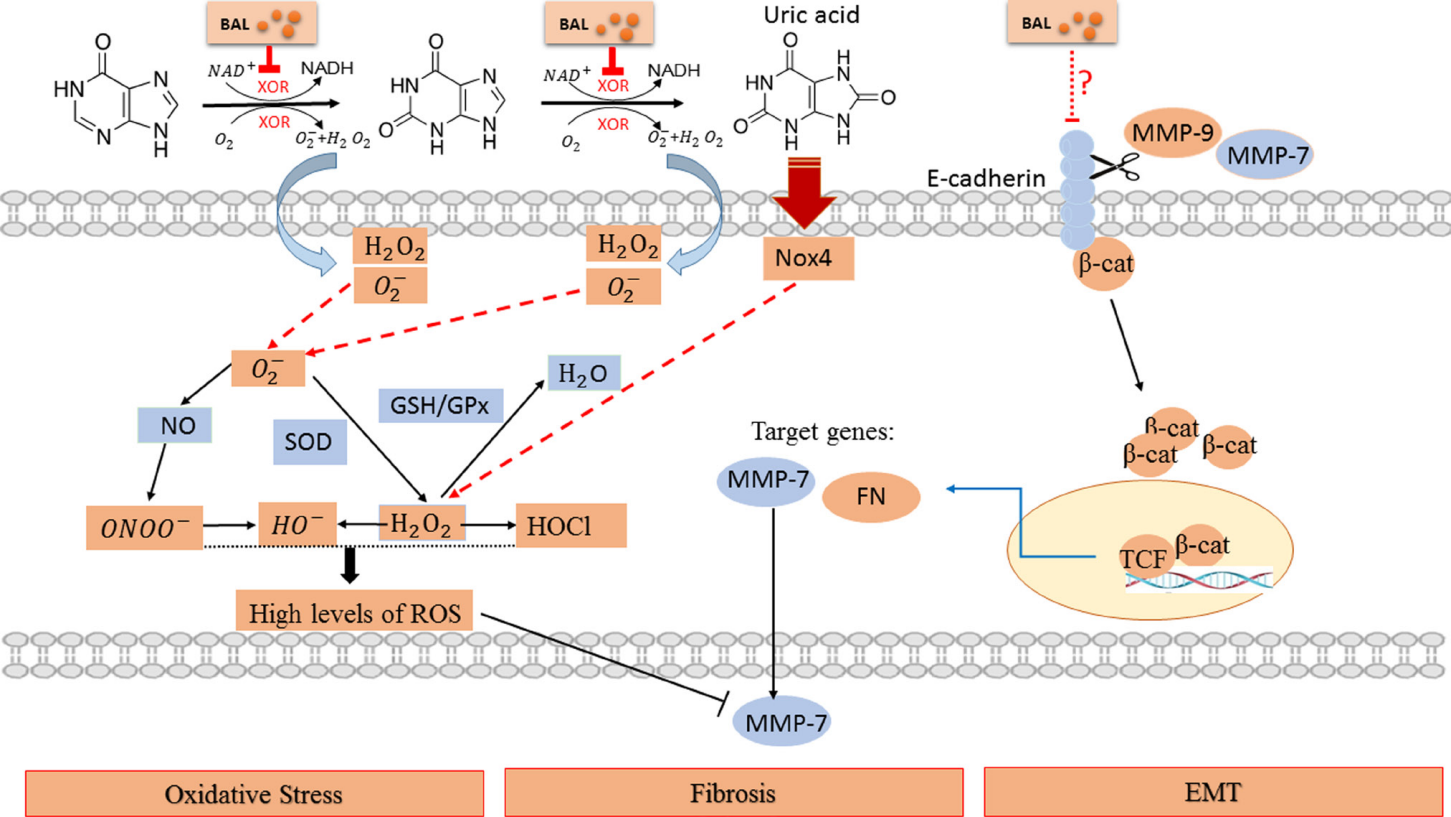
Schematic diagram of the potential pathway in hyperuricemia and interfered by baicalein Baicalein significantly suppressed the viability of XOR and remarkably prevented renal dysfunction, ameliorated kidney fibrosis, alleviated EMT and oxidative stress in hyperuricemia mice. Please see the text for more details.

Reactivating and hyperactive β-catenin signaling is detrimental in glomeruli and tubules. Moreover, genetic and pharmacologic activation of β-catenin may cause proteinuria [57, 58]. Accordingly, we have confirmed the activation of β-catenin and proteinuria in our hyperuricemia mice. MMP-7 and MMP-9 were also involved in β-catenin signal. They can promote degradation of E-cadherin, and the process leads to β-catenin release and activation [59, 60]. At the same time, the stabilization and nuclear translocation of β-catenin stimulated the transcription of target genes in kidney, including fibronectin and MMP-7 itself as well [61]. As only MMP-9 level was elevated, we thought that maybe MMP-9 played a more important role in this pathway. Based on previous reports, the decreased MMP-7 level might be concerned with the overproduction of ROS (Figure 7).

Meanwhile, we found an increased expression of α-SMA and a decreased level of E-cadherin, which indicated EMT process. EMT is a major source of fibroblasts in tissue fibrosis and MMPs are part of its regulation [52]. Both MMP-7 and MMP-9 could induce EMT in renal tubular cell cultures through cleavage of the epithelial cell marker E-cadherin [53, 62]. In our work, baicalein alleviated fibrosis and EMT through above pathways effectively. Therefore, our studies highlight that baicalein can alleviate fibrosis and EMT possibly through MMP-9/β-catenin signaling and MMP-7 in hyperuricemic nephropathy.

In summary, we are the first to demonstrate that baicalein can attenuate development of hyperuricemia-induced nephropathy. This beneficial effect may be attributed to XOR inhibition, NADPH oxidase-dependent and XOR-dependent ROS elimination, EMT moderation and fibrosis alleviation. Thus, baicalein may be developed as a candidate drug for hyperuricemia and hyperuricemia related other diseases.

## MATERIALS AND METHODS

### Ethics statement

All procedures in this study were carried out in strict accordance with the recommendations of the Guide for the Care and Use of Laboratory Animals of China Association for Laboratory Animal Science. All animal care protocols were approved by the Animal Care Committee of Peking University Health Science Center. All sacrifices were performed under pentobarbitone anesthesia, and every effort was made to minimize animal suffering.

### Baicalein

Baicalein is kindly provided by Topscience Bio-Pharm Technology. The compound's average molecular weight is approximately 270, as determined by high-performance steric exclusion chromatography analysis. In our experiments, baicalein was dissolved in sodium carboxymethyl cellulose (CMC) for animal treatment.

### Molecular docking

To simulate the binding modes of baicalein with two receptors: rat XOR (pdb entry: 1WYG) and human XOR (pdb entry: 2E1Q), molecular docking studies were performed via inserting baicalein into corresponding binding sites in crystal structures. The structure of baicalein was built using a 2D/3D editor sketcher in Catalyst software and the energy has been minimized. Parameters of docking were set in default. 20 conformations per calculation were generated via molecular docking and the best complex was chosen by docking total score.

### SPR biosensor analysis

The binding affinity of baicalein to XOR *in vitro* was assayed using the SPR-based Biacore 3000 instrument (Biacore AB, Uppsala, Sweden). XOR protein (molecular mass, 160 kDa) was purchased from Sigma-Aldrich. The XOR protein was immobilized on a CM5 sensor chip according to the standard procedures. The data were collected at a constant HBS-EP flow rate of 30 μl/min at 25°C. Baicalein was diluted into the running buffer to create a series of concentrations from 12.5 μM down to 0.78125 μM. The samples were injected into the channels at a flow rate of 30μl/min and the binding responses were continuously recorded in RU. The association (K_on_) and dissociation (K_off_) rate constants and the equilibrium dissociation constant (K_D_= K_off_) were calculated using BIA evaluation software version 3.1 (Biacore) with 1:1 Langmuir binding fitting model applied.

### Hyperuricemia mouse model

Male ICR mice (8 weeks -10 weeks old) weighing 20 g–22 g were purchased from the Animal Center of Peking University Health Science Center. The mice were acclimated to this environment for 7 d before experiments. The mice were divided randomly into four groups: the sham-operated group; the sham-operated baicalein (50 mg/kg)-treated group; the hyperuricemia group; and the hyperuricemia-baicalein (50 mg/kg)-treated group. The hyperuricemia mouse model was established by peritoneal injection of a mixture of hypoxanthine (0.3 g/kg) and potassium oxonate (0.3 g/kg) daily consistently for 2 weeks. In order to analyze the prevention efficacy of baicalein, it was orally injected for 21 days until sacrifice, including 7 days pretreatment. Animals treated with CMC alone were used as controls. After that, the animals were euthanized and the kidneys and livers were collected for protein analysis and histologic examination. Blood was also taken for the measurement of serum uric acid, BUN, creatinine, and other biochemistry indices. Twenty-four–hour urine samples were collected in metabolic cages at the last day for determination of urinary levels of protein.

### Assessment of UA, renal function, and other biochemistry indices

Serum UA, creatinine, BUN and urine UA were determined by commercial kits (NJJC Bio) according to the manufacturer's instructions. Urinary osmolality was measured using freezing point depression (Micro-osmometer, FISKER ASSOCIATES Q23). Microal buminuria was tested with ELISA kit (Wuhan Xinqidi Biological Technology Co., Ltd.).

### Assessment of serum activity of XOR

Serum activity of XOR was examined according to the protocol provided by the manufacture (NJJC Bio).

### Western blot analysis

Total protein was extracted using RIPA lysis buffer, and equal amounts of proteins were subjected to 8% SDS-PAGE and then transferred to polyvinylidene difluoride membranes (Millipore Corp., MA, U.S.A). The membranes were blocked and then incubated in primary antibodies against GAPDH (ABclonal Technology, China), fibronectin, MMP-7, MMP-9, Nox4 (ABcam), β-catenin (Epitomics), E-cadherin (Bioworld, China), α-SMA (Huaxing Bio., China) overnight at 4°C with gentle agitation, followed by incubation in the goat anti-rabbit IgG or goat anti-mouse IgG (Santa Cruz) labeled secondary antibody for 45 min at room temperature. Three 10-min washes in TBST were performed after secondary antibody labeling. The blots were developed with an ECL plus kit (Amersham Biosciences). The images were scanned with an Epson scanning system, and the data were analyzed with Quantity-one software. The data are expressed as the values relative to the sham or control value. When probing for multiple targets, stripping and re-probing a single membrane was needed. The membrane was incubated in stripping solution (Pplygen) for 20 min at room temperature with gentle agitation, followed by a 5 min wash in TBST. The membrane was blocked and then incubated in another primary antibody. Then the procedures above were conducted.

### Masson trichrome staining and periodic acid–schiff staining

Formalin-fixed kidneys were embedded in paraffin and prepared in 6 - μm - thick sections. Masson trichrome staining was performed according to the protocol provided by the manufacture (Sigma-Aldrich) to evaluate renal fibrosis, and Periodic acid–Schiff was conducted to test general histology.

### Statistical analyses

All the experiments were performed at least three times. All results are represented as the mean ± SEM. Data involving only two groups was analyzed by *t-test*. A *p-value* of < 0.05 was considered to be statistically significant difference for all tests.

## Abbreviations

UA: uric acid
CKD: chronic kidney disease
XOR: xanthine oxidoreductase
SPR: Surface Plasmon Resonance
EMT: epithelial-mesenchymal transition
STEMI: ST-segment elevation myocardial infarction
SOD: superoxide dismutase
GSH: reduced glutathione
GPx: glutathione peroxidase
Nox4: NADPH oxidase 4
MMPs: matrix metalloproteinases
α-SMA: α-smooth muscle actin
ROS: reactive oxygen species
ECM: extracellular matrix
CMC: carboxymethyl cellulose
OH^–^: hydroxyl anion
HOCl: hypochorous acid
